# Evaluation of Recombinant SAG1 Protein for Detection of *Toxoplasma gondii* Specific Immunoglobulin M by ELISA Test

**Published:** 2012

**Authors:** N Jalallou, M Bandehpour, H Khazan, A Haghighi, B Kazemi

**Affiliations:** 1 Dept. of Medical Laboratory Science, School of Medicine, Army Medical Science University, Tehran, Iran; 2 Dept. of Biotechnology, Shahid Beheshti University of Medical Sciences, Tehran, Iran; 3 Dept. of Medical Parasitology and Mycology, School of Medicine, Shahid Beheshti University of Medical Sciences, Tehran, Iran; 4 Cellular and Molecular Biology Research Centers, Shahid Beheshti University, of Medical Sciences, Tehran, Iran

**Keywords:** *Toxoplasma gondii*, Recombinant SAG1, IgM, ELISA

## Abstract

**Background:**

Toxoplasmosis is a serious disease in immunocompromised patients and pregnant women. Differentiation of acute and chronic infection is a major challenge in serodiagnosis of the disease. Since the aim of this study was to assess the diagnostic utility of recombinant SAG1 (rec-SAG1) for the detection of *Toxoplasma*-specific IgM antibodies in human sera, by an enzyme-linked immunosorbent assay (ELISA).

**Methods:**

The purified recombinant protein SAG1 was applied in house ELISA test and the ability of it in binding to specific immunoglobulin M in 30 serum samples of acute infected patients was evaluated. The results obtained by assays with the recombinant SAG1 and standard commercial assays were compared.

**Results:**

The sensitivity and specificity of in house ELISA compared to a standard commercial ELISA (com-ELISA) were 80% and 90%, respectively.

**Conclusion:**

It was concluded that the rec-SAG1 could be an alternative marker for detection of anti *Toxoplasma*-specific IgM and diagnosis of acute infection.

## Introduction

*Toxoplasma gondii* is an opportunistic pathogen, which is generally asymptomatic in immunocompetent individuals. In contrast, parasite transmission during pregnancy can result serious complications in fetal and neonatal (1). Also, toxoplasmic encephalitis has been reported as a major cause of death in HIV-AIDS patients (2). Therefore, using of specific and sensitive methods could be an important step towards treatment and prevention of toxoplasmosis. At present the existing tests have differ ability for detection of IgG and IgM antibodies in serums of infected persons; in most cases, they are based on soluble antigens of tachyzoite (3, 4). Owing to the intrinsic restrictions of such antigens that was applied in serological tests, purified recombinant antigens could be an appropriate choice for detection of serum antibodies (5). The surface antigens of the parasite are taken into consideration by most of the researchers, as they are the key of successful parasite entry into host cells (6). Among them, the surface antigen-1 (SAG-1) is a 30kD glycoprotein and could be detected in tachyzoite and sporozoite stages; has been proven to be a superior antigen for improving diagnostic tests or subunit vaccines (7).

One of the most important serological markers to identify acute toxoplasmosis is the presence of *Toxoplasma*-specific IgM antibodies and SAG1 is one of the first antigens recognized by IgM during acute infection (8). In spite of the absolute advantages of using recombinant SAG-1 in an enzyme-linked immunosorbent assay (ELISA) to detect *Toxoplasma*-specific IgG antibodies in human sera, there are different results about usefulness of this antigen in recognition of IgM specific antibodies (5, 9). Therefore the objective of this study was to evaluate the diagnostic utility of rec-SAG-1 by home-made ELISA in compression to commercial ELISA for detection of *Toxoplasma*-specific IgM antibodies in human serum samples.

## Materials and Methods

### Recombinant antigen

SAG-1 gene (accession number EF 140712) was used (10). The construction of the recombinant expression plasmid pET32a- SAG1 was carried out based on previous author publication (11). Briefly, the recombinant antigen was expressed in BL21 (DE3) pLysS competent bacterial cells as a fusion protein supplied with an N-terminal thioredoxin coding sequence and 6 His X6 tags at both ends. Then, rec-SAG1 was purified by Ni-affinity chromatography, the eluted fraction was dialyzed against PBS and the protein concentration was measured by Biophotometer.

### Serum Samples

A total number of 70 serum samples were used in this study. Sera were collected from diagnostic laboratories in Tehran and the Parasitology Department of Tehran University of Medical Sciences. Despite evaluation of sera in origin laboratories by different methods (IFA, ELFA, Chemiluminescence and ELISA) all of them were reexamined with a commercial ELISA kit (Euroimmun, Lubeek, Germany). Only serum samples that were positive or negative in both steps were used in the recombinant-ELISA (rec-ELISA).

### Rec- ELISA

The optimum antigen, serum concentration and conjugate dilution were determined by initial checkerboard titration. Purified rec- SAG-1 was diluted to the optimized concentration of 6 µg per ml in 50 mM carbonate- bicarbonate buffer (pH 9.6). One hundred µl of diluted r-SAG1 was coated to separate wells of flat bottomed polystyrene microplates (Greiner Bio One, Germany) at 4°C overnight. Wells were washed three times (PBS, pH 7.2, 0.05% Tween 20), and then blocked for 2 hours at room temperature with blocking solution (1% BSA and 0.05% Tween 20 in PBS). After repetition of washing stage, 100 µl of serum samples diluted 1:100 in blocking solution, were added into the wells and stored for 1.5 hours at room temperature. For each serum sample, the assay was done in duplicate, and average values were calculated. After three times washing, bound human IgM was detected by adding 100 µl Polyclonal goat anti- human IgM horseradish peroxidase conjugate (Abcam), 1:10000 in blocking solution to each well. The plates were incubated for one hour at room temperature, and then were washed like before. One hundred µl of the Ortho phenylene Diamine (OPD; Dako, Produktionsvej, Denmark) substrate solution (8 mg of OPD were dissolved in 12 ml of distilled water and 4 µl H2O2 prior to use) were added to each well. After 10 min, the color development reaction was stopped by adding 50 µl of 2N sulfuric acid and the color intensity was read at 490nm. The cut off for these assays was 2 standard deviation plus mean of the negative sera. Relative sensitivity and specificity were calculated as described by Aubert et al. (5)

## Results

**Fig. 1:**
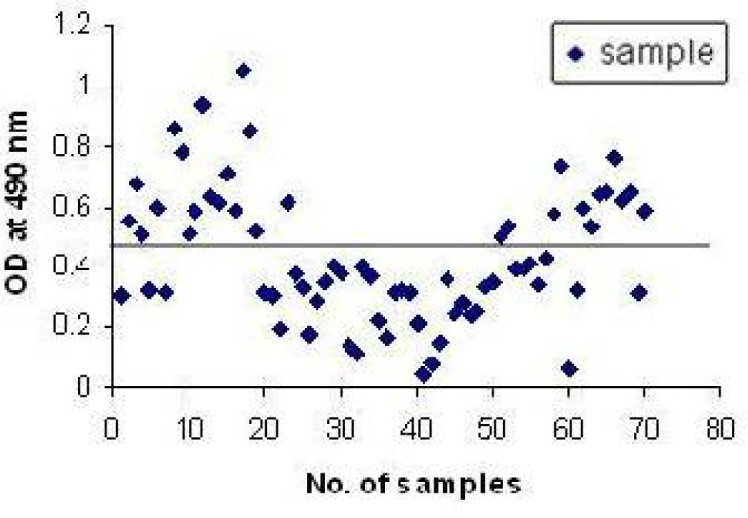
Profile of the antibody response in the IgM rec-ELISA of human sera using the rec-SAG1 protein (cut-off value: 0.478)

In order to determine cut off, purified rec-SAG1 protein was coated in the IgM rec-ELISA and tested with 20 serum samples that were negative for *Toxoplasma* IgG and IgM antibodies. The assay cut off was 2SD from the mean of negative population (mean=0.27, SD=0.104, cut off =0.478) (Fig. 1).

The immunoreactivity of rec-SAG1 was determined for 30 *Toxoplasma* IgM-positive sera and 40 *Toxoplasma* IgM and IgG negative sera, the results were summarized in Table 1.

**Table 1:** Comparison of rec-ELISA and com-ELISA in detection of IgM antibodies to *T. gondii*

		IgM com-ELISA		
		Positive	Negative	Total
*IgM rec-ELISA	Positive	24	4	28
	Negative	6	36	42
	Total	30	40	70

* rec-SAG1 was used as antigen

The sensitivity and specificity of designated rec-ELISA in comparison to the results obtained with com- ELISA was 80% (24/30) and 90% (36/40) respectively.

## Discussion

To date, many studies have been done to evaluate the ability of recombinant antigens (r-antigens) to bind *T. gondii*-specific antibodies (5, 12–15). Using recombinant antigens allow more precise standardization of the tests and allow designing new assays that can easily distinguish acute from chronic infection. In general, only a few reports explored the diagnostic utility of IgM rec-ELISA especially employing rec-SAG1 (8, 13 and 15).

SAG1 has been proven to be a valuable diagnostic tool, induces an intense immune response (11) and was conserved in many strains of *T. gondii* (16). Most of the time, detection of *Toxoplasma*- specific IgM could confirm acute infection. Therefore in the present study, we investigated IgM specific- *Toxoplasma* antibodies directed against rec-SAG1.

There are contradictory opinions about diagnostic utility of rec-SAG1 for *T. gondii*-specific IgM antibody. Aubert et al. (5) reported that the p30 antigen of *Toxoplasma* was recognized only 7 sera from 89 serum samples with a serological profile consistent with an acute Toxoplasmosis. In this experience, IgM assay with rec-SAG1 antigen in regarding to Aubert studies was relatively display a good performance, and could diagnose 80% of the infected patients (24/30). In another study that was carried in children at risk of congenital infection, ROC curves for the IgM capture immunoassay using the rec-SAG1 shows the best performance, in contrast to employ MIC2a, MIC2b and MIC3 antigens. The rec-SAG1 could detect 23 from 35 sera of infected infants (66%) (8); in comparison, we could get better results.

There are some explanations for these well-known differences: Most important of them, may be the variant presentation of this very complex molecule epitopes that was occurred in the preparation in different studies (17). As SAG1 is a highly conformational antigen and frequently expression in *E. coli* is in an insoluble form, therefore lose its immunoreactivity owing to incorrect folding (18, 19).

In the present study, rec-SAG1 as a soluble form of the fusion protein was expressed by using pET32a expression vector. Consequently, the constructed vector and the optimized expression situations, could improve diagnostic ability of rec-SAG1.

In spite of these differences, our findings were supported the results of Harning et al. opinion that rec-SAG1 is an appropriate marker for use in diagnostic systems to distinguish anti SAG1-specific IgG and IgM antibodies (13). Furthermore, we believed that many more samples have to be examined to confirm these results.

## Conclusion

It seems that rec-SAG1 could be a good marker for detection of anti *Toxoplasma*- specific IgM and diagnosis of acute infection, especially if it is used in combination with other tests, like IgG avidity.
